# Supplementary material for: Physicochemical, Nutritional and In Vitro Antidiabetic Characterisation of Blue Whiting (Micromesistius poutassou) Protein Hydrolysates
Source: Mar Drugs. 2021 Jul 2;19(7):383. doi: 10.3390/md19070383 (PMC8304566; doi:10.3390/md19070383)

**Figure S1:** Reversed phase ultra-performance liquid chromatography profiles of blue whiting (*Micromesistius poutassou*) soluble protein hydrolysates (BW-SPHs) pre- and post-simulated gastrointestinal digestion (GI)

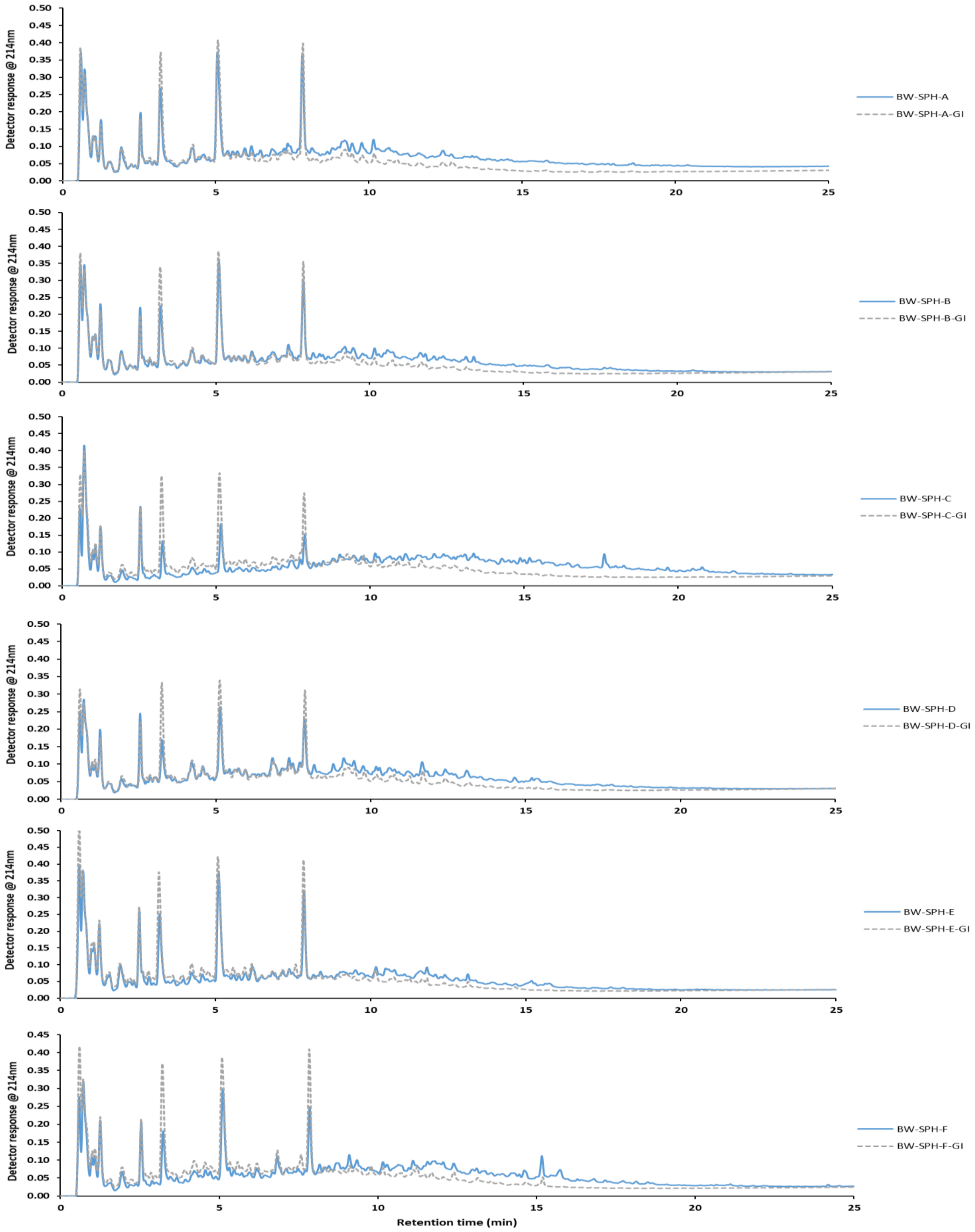

**Figure S2:** Effect of (A) blue whiting (*Micromesistius poutassou*) soluble protein hydrolysates (BW-SPH-A-F) and (B) their simulated gastrointestinal digestion (SGID) samples on cellular viability of BRIN-BD11 cells, measured using the (3-(4,5-dimethylthiazol-2-yl)-2,5-diphenyltetrazolium bromide (MTT) assay. Cells were co-incubated with hydrolysates prepared at 2.5 mg/ml in 5.6 mM glucose supplemented Krebs Ringer bicarbonate buffer for 1 h, followed by co-incubation with 5 mg/ml MTT solution for 1 h. Cells were lysed immediately after MTT solution removal with DMSO and the absorbance was measured at 570 nm. Values are represented as mean ± S.E.M. (n=4). ). Glucagon-like peptide-1 (GLP-1; $10^{-6}$ M) was used as a positive control. ***p<0.001 versus vehicle-only control.

(A)

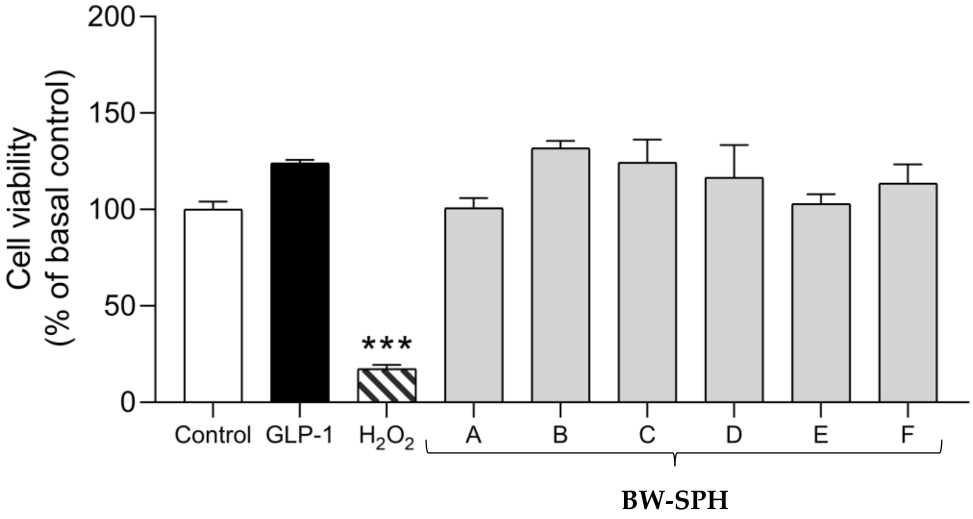


(B)

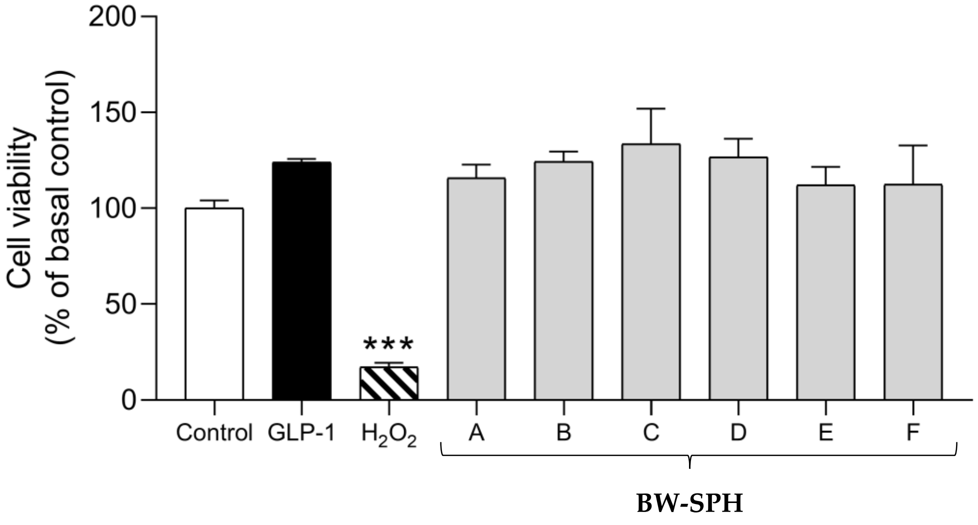

Supplement: Supplementary file 1 [file marinedrugs-19-00383-s001.zip › marinedrugs-1262376-supplementary.pdf]
